# Serum Autoantibodies in Patients with Dry and Wet Age-Related Macular Degeneration

**DOI:** 10.3390/jcm12041590

**Published:** 2023-02-17

**Authors:** Christina A. Korb, Sabine Beck, Dominik Wolters, Katrin Lorenz, Norbert Pfeiffer, Franz H. Grus

**Affiliations:** Department of Ophthalmology, University Medical Center, Johannes Gutenberg-University, 55131 Mainz, Germany

**Keywords:** age-related macular degeneration (AMD), immunoreactivities, antigen microarray, serum autoantibody profile

## Abstract

Background: To assess the serum autoantibody profile in patients with dry and exudative age-related macular degeneration compared with healthy volunteers to detect potential biomarkers, e.g., markers for progression of the disease. Materials and Methods: IgG Immunoreactivities were compared in patients suffering from dry age-related macular degeneration (AMD) (*n* = 20), patients with treatment-naive exudative AMD (*n* = 29) and healthy volunteers (*n* = 21). Serum was analysed by customized antigen microarrays containing 61 antigens. The statistical analysis was performed by univariate and multivariate analysis of variance, predictive data-mining methods and artificial neuronal networks were used to detect specific autoantibody patterns. Results: The immunoreactivities of dry and wet AMD patients were significantly different from each other and from controls. One of the most prominently changed reactivity was against alpha-synuclein (*p* ≤ 0.0034), which is known from other neurodegenerative diseases. Furthermore, reactivities against glyceraldehyde-3-phosphat-dehydrogenase (*p* ≤ 0.031) and Annexin V (*p* ≤ 0.034), which performs a major role in apoptotic processes, were significantly changed. Some immunoreacitvities were antithetic regulated in wet and dry-AMD, such as Vesicle transport-related protein (VTI-B). Conclusions: Comparison of autoantibody profiles in patients with dry and wet AMD revealed significantly altered immunoreactivities against proteins particularly found in immunological diseases, further neurodegenerative, apoptotic and autoimmune markers could be observed. A validation study has to explore if these antibody pattern can help to understand the underlying differences in pathogenesis, evaluate their prognostic value and if those could be possibly useful as additional therapeutic targets.

## 1. Introduction

Age-related macular degeneration (AMD) is a leading cause of irreversible visual impairment and severe vision loss in developed countries, classified as early stage with medium-size drusen and retinal pigmentary changes to late neovascular (wet or exudative AMD) or atrophic stages [1,2]. With trends towards increased life expectancy in the industrialised world, the number of people suffering from AMD is projected to reach 288 million in 2040, therefore AMD represents a substantial, global healthcare burden [3,4]. The etiology of AMD has multi-factorial, old age, cigarette smoking, cardiovascular risk factors and environmental, nutritional and genetic risk factors which contribute to disease pathogenesis [4,5]. Several studies reported the involvement of anti-retinal autoantibodies in ocular disorders, such as non-infectious uveitis [6], paraneoplastic and autoimmune retinopathy [7,8,9], retinitis pigmentosa [10,11], myopic macular degeneration [12] and age-related macular degeneration (AMD) [13,14,15,16,17,18,19]. Serum autoantibodies that bind retinal proteins have been detected in AMD patients in much higher frequencies than age matched controls [19,20,21,22]. AMD is a complex disease and these studies support the growing evidence that an immunological impact and inflammatory factors perform important roles in the pathogenesis of AMD. Accordingly, in a previous study, we could demonstrate a significant difference in IgG antibody patterns against retinal antigens between patients with “wet” AMD and healthy volunteers [23]. The differences were expressed by up- and down-regulations of antigen–antibody reactivities in the serum of AMD patients pointing to a shift in autoimmunity, including a possible loss of protective antibody functions. Until now the relevance and the role of autoantibodies in the pathogenesis of AMD is speculative and it is unclear if antibodies perform a causative role during the pathogenesis of AMD or appear as secondary effects during the disease’s progression.

The present study will point out the immunoproteomic differences between wet and dry AMD patients. The analyses will give evidence on up- and down-regulations of autoantibodies against retinal antigens and can possibly give insights into the panel of optimal serum markers of disease activity and future therapeutic mechanisms. After all, it could give valuable hints for a possible way towards an early detection of the disease or towards a possible immunoproteomic shift by prognostic biomarkers and towards a personalized medicine targeting specific pathways in the early stage.

## 2. Materials and Methods

Seventy subjects were recruited for this study, which was carried out in the Department of Ophthalmology, University Medical Center, Mainz, Germany. Written informed consent was obtained from all patients and the control group before study recruitment. The study had institutional review board/ethics committee approval, and the trial was undertaken in accordance with the Declaration of Helsinki. 

Inclusion criteria for participation in this study were the following: age ≥ 50 years, dry AMD in at least one eye in the dry AMD group (*n* = 20), in the neovascular AMD groups, participants with treatment-naive predominantly classic, minimally classic and occult lesions were eligible (*n* = 29) and age-matched healthy controls (*n* = 21). Table 1 shows the descriptive statistics of the study groups. Exclusion criteria included ophthalmic surgery or laser treatment within three months prior to inclusion in the study, diabetic retinopathy, retinal branch or central vein occlusion, current steroid medication, glaucoma, pathologic myopia, history of allergy to fluorescein, history of allergy to ranibizumab and current ocular or periocular infections.

Best-corrected visual acuity using Snellen charts at baseline and at every follow-up were recorded. Fluorescein angiography was used for diagnosis and spectral-domain OCT imaging (Spectralis OCT, Heidelberg Engineering, Heidelberg, Germany) was used for diagnosis and follow-up if needed. A 15 mL blood sample was drawn at baseline.

A correlation analysis was carried out in advance to exclude an influence of age on the antibodies. A significant effect could only be determined for Glial fibrillary acidic protein (GFAP), which is not one of the significant antibodies. For the remaining antibodies, age has no influence on the result. Therefore, the larger range in the control patients is remarkable, but not relevant for the further statistics. A possible reason for the slightly younger age of the patients in the control group could be the fact that other eye diseases, which served as exclusion criteria, such as diabetic retinopathy, retinal branch or central vein occlusion or glaucoma, occur more frequently in older age.

### 2.1. Seldi-TOF Analysis

The analysis of antigen-antibody profiles can be done in a reliable and sensitive manner using a developed proteomics technology: protein G Dynabeads combined with a ProteinChip system based on SELDI-TOF (surface enhanced laser desorption/ionization-time of flight) mass spectrometry (MS) [24]. The magnetic beads are designed to capture immunoglobulins [25] via a cell wall component, binding a wide range of IgG antibodies during incubation with various body fluids, such as sera. During a subsequent incubation with homogenized antigens, it is possible to capture relevant antigens by secondary binding to the antibodies. After elution antigens can be analysed by SELDI-TOF MS using ProteinChips with different, separating chip surfaces, e.g., cationic and anionic exchangers, hydrophobic surfaces and metal-ion affinity-chromatographic surfaces. Resulting mass spectra can be statistically analysed and compared to gain significantly higher or lower antigen–antibody reactivity peaks according to the study groups. The identification of potential biomarkers was performedusing highly sensitive MALDI-TOF/TOF (matrix assisted laser desorption/ionization–time of flight) MS (for more details see below).

### 2.2. Protagen Arrays

For the analysis of antibody patterns and identification of potential autoantibody biomarker candidates we chose a highly sensitive antigen microarray, which is a promising approach in this field of interest. This method has already been successfully used for the discovery of autoantibodies targeting prostate cancer specific biomarkers [8] and to screen sera of patients with, e.g., different pathological subtypes of multiple sclerosis or autoimmune hepatitis for autoreactive antibodies [26,27]. To screen autoantibody reactivities in study, sera was used as an advanced high density microarray approach. The sera of patients before treatment with ranibizumab(*n* = 10) were compared with the sera of the same patients after treatment with ranibizumab(*n* = 10). Two pools of ten sera were created for each group, which were incubated on nitrocellulose-coated slides (Grace Bio-Labs, Bend, OR, USA) with 3800 immobilized randomly selected human proteins from the UNIclone^®^ library (UNIchip^®^, Protagen, Dortmund, Germany) as described below. Incubation and washing steps were performed at 4 °C on an orbital shaker (Micromix 5, DPC, Los Angeles, CA, USA). Slides were covered with one-pad FAST-frame hybridization chambers (Whatman, Maidstone, UK) and blocked with PBS (Phosphate Buffered Saline, Invitrogen®, Carlsbad, USA) containing 0.5% BSA (Sigma-Aldrich, Steinheim, Deutschland) for one hour. Afterwards slides were washed three times ten minutes each time with PBS containing 0.5% Tween 20 (PBS-T, ICN Biomedicals, Meckenheim, Deutschland). Patients sera were diluted 1:375 in PBS and incubated on the Protagen-Slides overnight. After three washing steps with PBS-T, each time for ten minutes, slides were treated with fluorescence labelled secondary antibody (1:500 diluted in PBS, goat anti-human IgG, Jackson ImmunoResearch Laboratories, West Grove, PA, USA) for one hour in the dark. After three final washing steps, two with PBS-T and one with HPLC-grade ultra-pure Water (Mallinckrodt Baker BV, Holland) (ten minutes each time) slides were dried under vacuum. By using a high sensitive laser microarray scanner 16-bit TIFF (Tagged Information File Format) were generated. Spot intensities were quantified with ImaGene Software (ImaGene 5.5, Biodiscovery, El Segundo, CA, USA, 2012). After data normalization to internal standards with algorithm provided by Protagen, group differences were calculated and compared.

For visualization of the resultant antigen-antibody complexes, slides were treated with a secondary fluorescence labelled antibody (Dylight 650, Pierce, Rockford, IL, USA) followed by confocal laser scanning. After data normalization spot intensities were compared and group differences were analysed.

### 2.3. Analysis

Blood samples were centrifuged at 1000× *g* for ten minutes and the supernatant was stored at −80 °C for subsequent analysis. Magnetic protein G beads (Dynal, Oslo, Norway) were incubated with the patient’s sera. After several washings, the patient’s antibodies were covalently bound to the beads using ethanolamine. The bead-antibody complexes were then incubated with homogenized retinal antigens. The antigens bound to the patient’s autoantibodies were be eluted, concentrated and analysed by SELDI time-of-flight (TOF) MS ProteinChips with two different chromatographic surfaces (CM10 cation exchange and H50 reversed phase). The samples were measured with a SELDI-TOF MS ProteinChip system (Bio-Rad, Hercules, CA, USA) on a PBS-IIc ProteinChip Reader. Raw data was transferred to CiphergenExpress 2.1 database software (Bio-Rad, Hercules, CA, USA) for workup and analysis. An in-house developed Proteomics Software Project (PSP) statistically evaluated the spectra using different statistical approaches to guarantee a high specificity and sensitivity of antibody patterns for the observed study groups. The PSP additionally searched for highly significant biomarkers directing a Statistical based analysis using above mentioned algorithms. The identification of biomarkers was performed by MALDI-TOF/TOF MS analysis (Bruker, Billerica, MA, USA). We aimed to generate at least eight highly specific biomarkers (significance level α = 0.05 and power (1 − ß) = 90%) for “wet” AMD.

Statistical calculations of sample sizes were based on experiences from previous studies (e.g., [28]): the calculated number of cases is sufficient to detect an effect on the serum antibody profiles, given a significance level α = 0.05 and power (1 − ß) = 90%. The statistical analysis demonstrated that the antibody composition against retinal antigens within sera change over time. A comparison to the control group showed if the modifications are beneficial, i.e., the serum compositions become more similar to the serum of healthy subjects or not. A subsequent biomarker identification using MALDI-TOF/TOF MS (Bruker, Billerica, MA, USA). revealed valuable hints on the systemic effects. After electrophoretic separation, proteins were be typically digested, crystallized on matrix and analysed on a MALDI target. The obtained peptide mass fingerprint data were exported into BioTools (Version 3.1, Bruker, MA, USA) and used for an internal Mascot database search (Matrix Science, London, England; Uniprot release 07, 2012), leading to protein identifications.

### 2.4. Antigen Microarrays

In this study, we used highly purified proteins, purchased at Sigma-Aldrich (Taufkirchen, Germany) and BioMol (Hamburg, Germany), as antigens. The antigen selection is based on previous autoantigen identifications in glaucoma patients by our group and survey of the literature related to identifications of autoantigens in autoimmune diseases and age-related macular degeneration. Antigens were diluted to 1 µg/µL with PBS buffer containing 1.5% Trehalose (ICN Biomedicals, Meckenheim, Deutschland) for optimal printing conditions. The spotting of antigens was performed with both a non-contact printing technology (sciFLEXARRAYER S3, Scienion, Berlin, Germany), based on piezo dispensing, and the commonly used pin based contact printing technique (OmniGrid100, Digilab Genomic Solutions, Ann Arbor, MI, USA). Results were comparatively evaluated for spot morphology and spot to spot variability. For printing of the whole set of study microarrays the piezo based spotting technique was used. Each antigen was spotted in triplicate onto nitrocellulose-slides (Oncyte, nitrocellulose 16 multi-pad slides, Grace Bio-Labs, Bend, USA). As a positive and negative control, we used mouse anti-human IgG/A/M (10 μg/μL) and spotting buffer. The spotting process was performed at RT and a humidity of 30%. A total of 1 nL of each antigen-dilution was applied onto the nitrocellulose surface by spotting four times 250 pl on exactly the same position. The accurateness of the spotting volume and the correct positioning of the droplets were monitored before and after the spotting process of each antigen using the sciDrop-VOLUME and auto drop detection software (Scienion, Berlin, Germany). 

Incubation and washing steps were performed at 4 °C on an orbital shaker (Titramax 100, Heidolph, Schwabach, Germany). Slides were covered with 16 pad FAST frame hybridization chambers (Whatmann, Maidstone, UK) and blocked with PBS containing 4% BSA for one hour. Afterwards slides were washed three times with PBS containing 0.5% Tween (PBS-T). Patient sera were diluted 1:250 in PBS and aqueous humour in a ratio of 1:10 in PBS. A total of 120 μL of these dilutions were randomly incubated on prepared antigen-slides overnight. After several washing steps with PBS-T, slides were incubated with a fluorescent Cy-5 labelled secondary antibody (1:500 diluted in PBS-T, goat anti-human IgG, Jackson ImmunoResearch Laboratories, West Grove, PA, USA) for one hour in the dark. Two washing steps with PBS-T were followed by two final washing steps with HPLC-grade water. All microarrays were air dried before scanning, using a microarray scanner (Affymetrix 428 TM Array Scanner, High Wycombe, UK). Generated 16-bit TIFF images (Tagged Information File Format) of slides were analysed using the Spotfinder 3.1.1 software (TM4, Dana-Faber Cancer Institute, Boston, MA, USA) [29,30]. Background subtraction was performed according to the formula: spot intensity = mean intensitySP − ((sumbkg − sumtop5bkg)/(number of pixelbkg − number of pixelstop5bkg)), where SP represents any spot, bkg the corresponding background and top5bkg the top five percent of background pixel. The coefficient of variance (CV) was calculated as follows: CV = SDSP3/meanSPX…SPn, where SDSP3 represents the standard deviation across three replicate spots of one antigen of one sample and meanSPX…SPn the mean of all spot intensities [31]

## 3. Results

IgG immunoreactivities were measured in three groups: controls, dry AMD and newly diagnosed treatment naive neovascular AMD.

In the present study, we first analysed the autoantibody patterns against retinal antigens in “wet” AMD sera samples and compared them to control sera samples and “dry” AMD sera samples by mass spectrometry (MS) approach. After successful de novo screening of immunoreactivities using MS-based approach and high density Protagen antigen microarrays, a customized antigen microarrays containing 61 antigens was built (Table 2). Each microarray contained each antigen as triplicate.

Analysis of immunoreactivities of IgG against these 61 antigens was performed in 70 samples.

### Comparison of Immunoreactivities in Dry and Wet AMD

**Figure 1:**
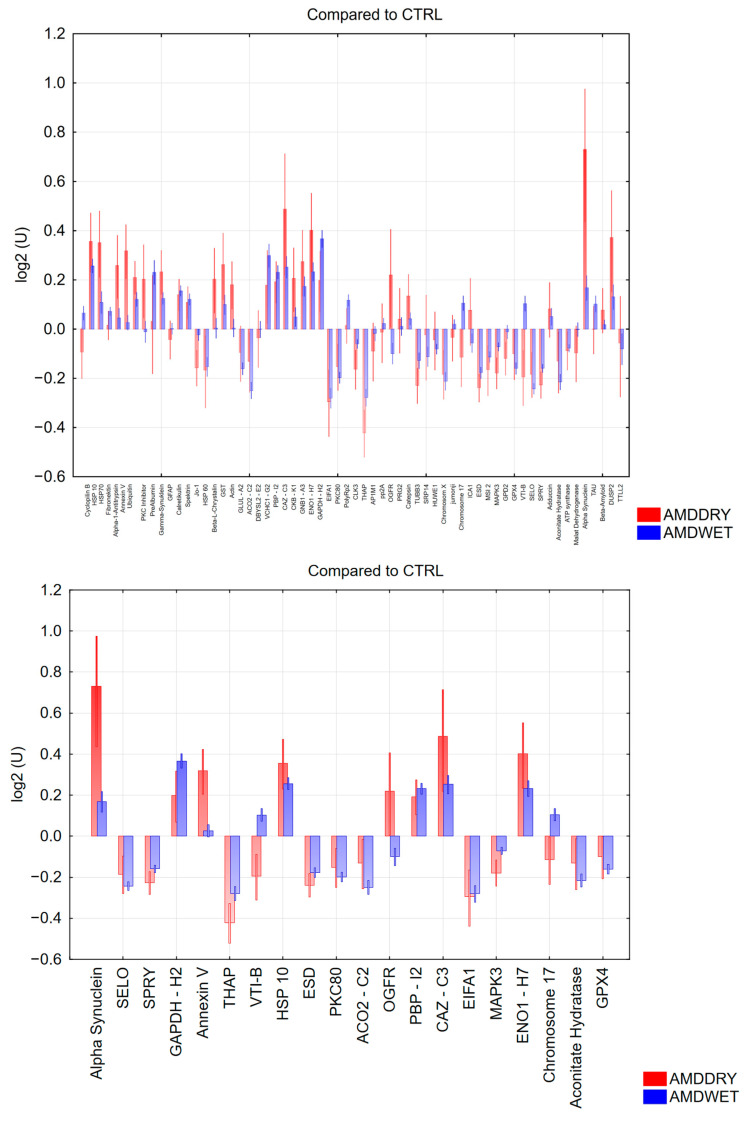
Comparison of immunoreactivities from dry AMD and wet AMD samples compared to CTRL. **Top**: all immunoreactivities; **bottom**: 20 most significant reactivities.

In a first step, the immunoreactivities were analysed in samples from dry AMD and wet AMD and compared to healthy control samples (Figure 1). Table 3 shows the results of ANOVA analysis and their corresponding *p*-values for the most-significant antigens. 

The immunoreactivities of dry and wet AMD samples are highly significantly different from each other and from controls. Although, as part of the natural autoimmunity also found in healthy subjects, complex antibody patterns against the tested antigens could show that the patterns in both AMD groups are changed. One of the most prominently changed reactivity is against alpha-synuclein. Alpha-synuclein is known from other neurodegenerative diseases. Others are heat shock proteins (e.g., HSP10) and Annexin V, which performs a major role in apoptotic processes. Some others are antithetic regulated in wet and dry AMD, such as VTI-B, PBP-12 and OGFR.

Pathway comparison analysis revealed protein functions particularly found in immunological diseases, especially aconitase 2, which performs a role in citric acid cycle, furthermoreenolase 1, Annexin V, mitogen-activated protein kinase C (MAPK3) and Alpha-Synuclein. Mutations in Alpha-synuclein are associated with Parkinson’s disease, Alzheimer’s disease and several other neurodegenerative illnesses. Annexin 5 is a phospholipase A2 and protein kinase C inhibitory protein with calcium channel activity and a potential role in cellular signal transduction, inflammation, growth and differentiation.

Apart from the immunological markers, bio functions performing a major role in inflammation could be observed, e.g., Alpha-synuclein, aconitase 2, enolase 1 and GADPH (glyceraldehyde-phosphate dehydrogenase), which acts in apoptotic processes and is also known to perform a role in Alzheimer’s disease. Annexin V is proposed to have anti-apoptotic and anti-inflammatory functions, comparison of immunoreactivities in our study revealed higher reactivity in the dry AMD samples.

## 4. Discussion

In the present study, we first investigated differences in the immunoreactivities in dry and exudative AMD serum samples to gain insight into the pathogenesis and contribute to the understanding of the underlying disease mechanisms. In recent years, biomarkers have become major indicators of personalized medicine, in particular serological biomarkers for diagnosis, prognosis and response to treatment [20,32]. It is known that two-third of serum immunoglobulins in healthy individuals are natural occurring autoantibodies, so that complex profiles exist even in healthy individuals [33,34,35] and disease specific changes of circulating autoantibodies are known from several other diseases, e.g., glaucoma or Sicca syndrome [36,37,38,39,40]. Serum anti-retinal autoantibodies were detected at a much higher incidence in patients with early AMD than in individuals without AMD, suggesting their diagnostic value [16,17,20]. Moreover, it has been suggested that distinct autoantibody signatures may exist between early AMD, geographic atrophy and neovascular AMD [17,18]. However, the role of anti-retinal autoantibodies in the induction or progress of retinal degeneration is not well defined, although it has been shown that anti-retinal autoantibodies can develop on average 3–15 years prior to the first clinical signs [20,41].

Inflammation and oxidative stress have been proposed as central mechanisms in the pathophysiology of AMD [42]. In this study, one of the most prominently changed reactivity was against alpha-synuclein. Alpha-synuclein (α-syn) modulates retinal iron homeostasis and has implications for visual manifestations of Parkinson’s disease [43]. Alpha-synuclein is also widely distributed in the retina and previous studies suggested a role of the synuclein family, including α-syn, in retinal neurodegeneration and particularly during remodelling [44,45]. Ferroptosis is an iron-dependent regulated cell-death pathway and has been implicated in AMD pathogenesis [46,47]. In the recent study, immunoreactivity against α-syn was significantly upregulated in patients with dry AMD. The differences in immunoreactivities in the group of patients with dry and wet AMD compared to control may suggest that autoantibodies against α-syn participate in pathogenity of AMD. Furthermore, an analysis of antibody titre in patients with different stages of dry AMD might be valuable, especially if potential inactivation or removal of specific antibodies may have a monitoring or therapeutic effect in reducing the progression of dry AMD in the future. 

Annexin V is a phospholipid-binding protein with an important role in the regulation of apoptosis, it is highly expressed by vascular endothelial cells where it is thought to function antithrombolically [48]. Different expression levels have been reported for several annexins and a wide range of diseases, suggesting a potential use to determine disease progression and therapeutic monitoring [49]. A role for Annexin V was identified in the recognition and binding step of clearance phagocytosis, which is essential to retinal physiology [50]. Neovascular AMD was associated with altered gene expression in peripheral white blood cells, among others increased levels of AnnexinA5 mRNA transcripts were found and it was postulated that Annexin A5 levels may increase in AMD as part of a healing response [51]. Anti-Annexin A5 was upregulated in serum of patients with early to advanced AMD compared to control serum before and could contribute to AMD pathogenesis via an autophagy-mediated mechanism [18]. Furthermore, Annexin A1 and Annexin A4 were upregulated in the tears of patients with neovascular AMD, possibly indicating a disturbed proteostasis in AMD [52]. In our study, comparison of immunoreactivities revealed higher reactivity in the dry AMD samples, but immunoreactivity in neovascular AMD samples was also higher than in the control group, suggesting that in both groups anti Annexin A5 autoantibodies might contribute to the pathogenesis of AMD.

Glyceraldehyde-3-phosphate dehydrogenase (GAPDH) is a glycolytic enzyme and is also involved in regulating cell death, its role in the development of retinal diseases has been explored in previous studies [53]. GAPDH performs a significant role in the development of diabetic retinopathy and its progression [54]. Moreover, overexpression of GAPDH inhibited neurovascular degeneration after retinal injury [53]. In retinal pigment epithelial cells, GAPDH was differentially expressed after exposure to UVA radiation, possibly contributing to photoreceptor dysfunction [55]. Interestingly, autoantibodies against GAPDH in patients with autoimmune retinopathy were associated with disease severity [56]. In the present study, we found immunoreactivities of anti-GAPDH elevated in AMD patients compared to control with the most pronounced elevation in patients with neovascular AMD. Thus, anti-GAPDH autoantibodies especially in the group of patients with neovascular AMD could possibly contribute to the pathogenesis of induction of apoptosis and proliferation.

Autoantibodies against Opioid Growth Factor Receptor (OGFr) were antithetically regulated in patients with dry and wet AMD compared to controls, with an upregulation in patients with dry AMD. The Opioid Growth Factor (OGF), chemically termed [Met5]-enkephalin, is an endogenous pentapeptide that binds to OGFr, the pathway is responsible for homeostasis of cell replication and renewal [57]. OGF-OGFr axis performs a key role in the homeostasis of cornea and retina [58,59]. Depending on the duration of OGFr blockade, opioid antagonists, such as naloxone, are effective therapies for cancer, autoimmune diseases and complications associated with diabetes [60]. In mice, naloxone significantly reduced the progress of retinal AMD-like lesions, as naloxone modulates microglia accumulation and activation at the site of retinal degeneration [61]. In the present study, we found an antithetical regulation of anti-OGFr in patients with dry and neovascular AMD. These observations suggest that the OGF-OGFr axis might perform an important role in the pathogenesis of AMD and verification of the role of autoantibodies is desirable.

Strengths of the present study are the prospective nature and the analysis by customized antigen-microarrays containing 61 antigens after successful de novo screening of immunoreactivities using MS-based approach. Antigen-Microarrays allows for fast evaluation with high sensitivity of potential autoantibody biomarkers using small microliter sample volumes. Furthermore, an aged-matched control group with participants without age-related macular degeneration was included. Fluorescein angiography and spectral-domain OCT imaging were used for diagnosis in all patients.

Despite the strengths, our study has several limitations. First, the presence of immunoreactivities is neither sensitive nor specific for the diagnosis of age related macular degeneration. In general, it has to be elucidated whether the described autoantibodies perform a causative role in the pathogenesis of AMD or appear as secondary effects during the disease’s progression. Second, a group of patients with dry AMD was included without further evaluating different AMD stages. Subsequent studies are planned and required to evaluate immunoproteomic differences in different stages of the disease in order to possibly act as a diagnostic tool, to establish prognostic markers of disease activity and progression and to obtain therapeutic treatments. Third, this study elucidated immunoreactivities against 61 antigens, which were preselected after successful de novo screening of immunoreactivities using MS-based approach and high density Protagen antigen microarrays. Although we found statistically significant immunoproteomic differences in the group of patients with dry and wet AMD, natural occurring autoantibodies also exist in healthy individuals. Fourth, in this study, patients were not eligible if they fulfilled the stated exclusion criteria. Other comorbidities or factors, such as body mass index, cholesterol level, blood pressure and blood glucose level, were not evaluated in this study. Subsequent validation studies, including possible correlating individual comorbidities, could provide further valuable insights in the immunoproteomic profiles in patients with dry and exudative AMD a possible impact on diagnosis, progression and therapy.

In sum, our study provides huge differences in the immunoreactivities in both wet and dry AMD samples. The limitations described above highlight the need for standardised and appropriately designed studies, which are needed to explore if antibody patterns can help understand the underlying differences in pathogenesis of dry and exudative AMD and evaluate their prognostic or possibly therapeutic value.

## Figures and Tables

**Table 1:** Descriptive statistics of study groups.

Group	N (Sample Size)	Gender (m/f)	Age—Means	Age—Std.Dev.	Age—Minimum	Age—Maximum
CTRL	10	f	71.8	9.36453	54	82
CTRL	11	m	74.5	10.98511	55	90
17	14	f	80.9	8.54722	64	97
17	6	m	73.2	7.19491	63	82
18	20	f	81.0	7.93339	59	92
18	9	m	81.4	5.11503	72	88

CTRL = healthy volunteers, 17 = dry AMD, 18 = newly diagnosed wet AMD.

**Table 2:** List of antigens on customized microarray.

ID	MW [kDa]	Protein Name UniProt	Protein Name	Abbreviation in Study
P62937	18.0	Peptidyl-prolyl cis-trans isomerase A (Cyclophilin A)	Cyclophilin A human	Cyclophilin B
P61604	10.9	10 kDa heat shock protein, mitochondrial (Hsp10)	Chaperonin 10, Recombinant, Human	HSP 10
P00441	15.9	Superoxide dismutase [Cu-Zn]	Superoxide Dismutase from bovine erythrocytes	SOD
P02686	33.1	Myelin basic protein (MBP); Isoform 1	Myelin Basic Protein from bovine brain	MBP
P04792	22.8	Heat shock protein beta-1 (Heat shock 27 kDa protein; Hsp27)	Hsp27 Protein—Low Endotoxin	HSP 27
P08107	70.1	Heat shock 70 kDa protein 1A/1B (Hsp70.1/Hsp70.2)	Heat Shock Protein 70 from bovine brain	HSP 70
P02751	262.6	Fibronectin; Isoform 1	Fibronectin from human plasma	Fibronektin
P01009	46.7	Alpha-1-antitrypsin	α_1_-Antitrypsin from human plasma	Alpha-1-Antitrypsin
P08758	35.9	Annexin A5	Annexin V from human placenta	Annexin V
Q14694	87.1	Ubiquitin carboxyl-terminal hydrolase 10 (USP10)	Ubiquitin human	Ubiquitin
P49773	13.8	Histidine triad nucleotide-binding protein 1 (Protein kinase C inhibitor 1)	Protein Kinase C Inhibitor, Myristoylated	PKC Inhibitor
P02766	15.9	Transthyretin (Prealbumin)	Prealbumin from human plasma	PreAlbumin
O76070	13.3	Gamma-synuclein	γ-Synuclein human	Gamma-Synuklein
P14136	49.9	Glial fibrillary acidic protein (GFAP)	Anti-Glial Fibrillary Acidic Protein	GFAP
P27797	48.1	Calreticulin	Calreticulin from bovine liver	Calretikulin
P02549	280.0	Spectrin alpha chain, erythrocyte	Spectrin from human erythrocytes	Spektrin
P12081	57.4	Histidine-tRNA ligase, cytoplasmic (JO-1)	JO-1 human	Jo-1
P10809	61.1	60 kDa heat shock protein, mitochondrial (Hsp60)	HSP60 (human), (recombinant)	HSP 60
P53674	28.0	Beta-crystallin B1	β_L_-Crystallin from bovine eye lens	Beta-L-Chrystalin
P09211	23.4	Glutathione S-transferase P	Glutathione S-Transferase from bovine liver	GST
P68133	42.1	Actin, alpha skeletal muscle	Actin from bovine muscle	Actin
P15104	42.1	Glutamine synthetase	Glutamine synthetase	GLUL
Q99798	83.4	Aconitase 2, mitochondrial	aconitase 2, mitochondrial	ACO2
E5RFU4	18.3	Dihydropyrimidinase-like 2	Dihydropyrimidinase-like 2	DBYSL2
P09936	24.8	Ubiquitin carboxyl-terminal hydrolase isozyme L1 (UCHL1)	Ubiquitin carboxyl-terminal hydrolase isozyme L1	VCHC1
P30086	21.1	Phosphatidylethanolamine-binding protein 1	Phosphatidylethanolamine-binding protein 1	PBP
P00918	29.2	Carbonic anhydrase 2	Carbonic Anhydrase II	CAZ
P12277	42.6	Creatine kinase B-type	Creatine kinase B	CKB
P62873	37.4	Guanine nucleotide-binding protein G(I)/G(S)/G(T) subunit beta-1 (GNB1)	Guanine nucleotide-binding protein G(1)/G(S)/G(T) subunit beta 1	GNB1
P06733	47.2	Alpha-enolase	Alpha-Enolase	ENO1
P04406	36.1	Glyceraldehyde-3-phosphate dehydrogenase (GAPDH)	Glyceraldeyde (3-)phosphate dehydrogenase	GAPDH
P60842	46.2	Eukaryotic initiation factor 4A-I	Homo sapiens eukaryotic translation initiation factor 4A isoform 1 (EIF4A1) mRNA	EIFA1
A8K318	59.2	Protein kinase C substrate 80K-H	protein kinase C substrate 80K-H isoform 2 [Homo sapiens]	PKC80
Q68Y55	34.9	Poly(RC) binding protein 2	poly(rC) binding protein 2 isoform g [Homo sapiens]	PolyRp2
P49761	58.6	CDC-like kinase 3 (CLK3), transcript variant phclk3, mRNA	Homo sapiens CDC-like kinase 3 (CLK3); transcript variant phclk3; mRNA	CLK3
Q9P2Z0	28.4	THAP domain-containing protein 10	THAP domain containing 10 [Homo sapiens]	THAP
Q9BXS5	48.6	AP-1 complex subunit mu-1 (AP1M1)	Homo sapiens adaptor-related protein complex 1; mu 1 subunit (AP1M1); mRNA	AP1M1
P63330	35.6	Serine/threonine-protein phosphatase 2A catalytic subunit alpha isoform	protein phosphatase type 2A catalytic subunit alpha isoform [Mus musculus]	pp2A
Q9NZT2	73.3	Opioid growth factor receptor	Homo sapiens opioid growth factor receptor (OGFR). mRNA	OGFR
			Homo sapiens plasticity-related gene 2 (PRG2) mRNA	PRG2
P43235	37.0	Cathepsin K	cathepsin K preproprotein [Homo sapiens]	Catepsin
Q53G92	50.4	Tubulin beta-3 chain	Homo sapiens tubulin beta 3 (TUBB3) mRNA	TUBB3
P37108	14.6	Signal recognition particle 14 kDa protein	Homo sapiens signal recognition particle 14 kDa (homologous Alu RNA binding protein) (SRP14) mRNA	SRP14
Q7Z6Z7	481.9	E3 ubiquitin-protein ligase HUWE1; Isoform 1	HUWE1 protein [Homo sapiens]	HUWE1
			Homo sapiens chromosome X genomic contig. reference assembly	ChromosomX
Q96S16	36.9	JmjC domain-containing protein 8 (Jumonji domain-containing protein 8)	jumonji domain containing 8 [Homo sapiens]	jumonji
Q96N21	55.1	Uncharacterized protein C17orf56	Homo sapiens chromosome 17 open reading frame 56 (C17orf56). mRNA	Chromosome 17
Q96HG3	54.6	Islet cell autoantigen 1, 69 kDa	Homo sapiens islet cell autoantigen 1. 69 kDa (ICA1). transcript variant 2. mRNA	ICA1
P10768	31.5	S-formylglutathione hydrolase (Esterase D)	Homo sapiens esterase D/formylglutathione hydrolase (ESD)	ESD
P25325	33.2	3-mercaptopyruvate sulfurtransferase	mercaptopyruvate sulfurtransferase isoform 2 [Homo sapiens]	MSI 2
P27361	43.1	Mitogen-activated protein kinase 3; Isoform 1	Homo sapiens mitogen-activated protein kinase 3 (MAPK3); transcript variant 1; mRNA	MAPK3
P43304	80.9	Glycerol-3-phosphate dehydrogenase, mitochondrial (GPD2); Isoform 1	Homo sapiens glycerol-3-phosphate dehydrogenase 2 (mitochondrial) (GPD2); mRNA	GPD2
P36969	22.2	Phospholipid hydroperoxide glutathione peroxidase, mitochondrial (Glutathione peroxidase 4	Homo sapiens glutathione peroxidase 4 (phospholipid hydroperoxidase) (GPX4). transcript variant 1. mRNA	GPX4
B7Z4U7	65.1	Sec1 family domain containing 1, isoform CRA_b	vesicle transport-related protein isoform b [Homo sapiens]	VTI-B
Q9BVL4	73.5	Selenoprotein O	Homo sapiens selenoprotein O (SELO) mRNA	SELO
Q6PJ21	39.4	SPRY domain-containing SOCS box protein 3	SPRY domain-containing SOCS box protein SSB-3	SPRX
P35611	81.0	Alpha-adducin	adducin 1 (alpha) isoform c [Homo sapiens]	Adduccin
Q99798	85.4	Aconitate hydratase, mitochondrial	Aconitate Hydratase 2 (mitochondrial)	Aconitate Hydratase
P06576	56.6	ATP synthase subunit beta, mitochondrial	ATP synthase	ATP Synthase
P40926	35.5	Malate dehydrogenase, mitochondrial	Malat dehydrogenase	Malat Dehydrogenase
P37840	14.5	Alpha-synuclein	alpha-synuclein	Alpha Synuclein
P10636	78.9	Microtubule-associated protein tau	tau	TAU
P05067	86.9	Amyloid beta A4 protein (Alzheimer disease amyloid protein)	beta-amyloid	Beta-Amyloid
Q05923	34.4	Dual specificity protein phosphatase 2	DUSP2 dual specificity phosphatase 2 [*Homo sapiens*]	DUSP2
Q14166	74.4	Tubulin-tyrosine ligase-like protein 12	Homo sapiens tubulin tyrosine ligase-like family member 12 (TTLL12) mRNA	TTLL2

**Table 3:** ANOVA (Analysis of Variance) of the IgG immunoreactivities in wet AMD and dry AMD samples compared to controls.

ANOVA							
	CTRL-AV	CTRL-SE	AMDDRY-AV	AMDDRY-SE	AMDWET-AV	AMDWET-SE	ANOVA-*p*
Alpha Synuclein	6428	487	10.667	1974	7221	251	0.003
SELO	31.155	1755	27.412	1733	26.330	397	0.01
SPRY	24.905	1445	21.287	808	22.305	265	0.028
GAPDH—H2	11.571	821	13.277	1137	14.926	360	0.031
Annexin V	14.977	1821	18.675	1422	15.258	321	0.034
THAP	14.422	1575	10.767	715	11.889	284	0.044
VTI-B	22.121	1816	19.333	1493	23.773	512	0.065
HSP 10	16.099	1313	20.613	1716	19.235	385	0.071
ESD	29.416	1393	24.942	995	26.008	424	0.082
PKC80	23.592	1798	21.236	1396	20.575	335	0.082
ACO2—C2	19.238	1478	17.569	1462	16.185	376	0.089
OGFR	18.774	2383	21.865	3017	17.521	516	0.115
PBP—I2	21.243	1455	24.279	1422	24.944	467	0.119
CAZ—C3	5373	595	7534	1274	6402	196	0.148
EIFA1	26.612	2773	21.695	2045	21.915	612	0.15
MAPK3	28.505	1571	25.186	1110	27.123	315	0.15
ENO1—H7	19.297	1419	25.493	2798	22.685	594	0.15
Chromosome X reading frame 56	20.439	1587	18.881	1513	21.993	455	0.16
Aconitate Hydratase	20.584	1720	18.805	1622	17.738	391	0.163
GPX4	19.207	1159	17.929	1277	17.190	283	0.177

The table reveals the most significant antigens and according *p*-values.

## Data Availability

Not applicable.
